# An open-label, multicenter, phase Ib study investigating the effect of apalutamide on ventricular repolarization in men with castration-resistant prostate cancer

**DOI:** 10.1007/s00280-018-3632-6

**Published:** 2018-07-05

**Authors:** Bodine P. S. I. Belderbos, Ronald de Wit, Caly Chien, Anna Mitselos, Peter Hellemans, James Jiao, Margaret K. Yu, Gerhardt Attard, Iurie Bulat, W. Jeffrey Edenfield, Fred Saad

**Affiliations:** 1 Erasmus MC Cancer Institute, Dr. Molewaterplein 40, 3015 GD Rotterdam, The Netherlands; 2 Janssen Research & Development, 1400 McKean Road, Spring House, PA 19477 USA; 3 Janssen Research & Development BE, Turnhoutseweg 30, Beerse, Belgium; 4 Janssen Research & Development, 920 Route 202 South, Raritan, NJ 08869 USA; 5 Janssen Research & Development, 10990 Wilshire Blvd., Suite 1200, Los Angeles, CA 90024 USA; 6 Research Department of Oncology, UCL Cancer Institute, 72 Huntley Street, London, WC1E 6DD UK; 7 ARENSIA Exploratory Medicine’s Research Unit, The Institute of Oncology, 30 N.Testemitanu str., 2025 Chişinău, Republic of Moldova; 8 GHS Cancer Institute, 900 West Faris Road, Greenville, SC 29615 USA; 9 Centre Hospitalier de l‘Université de Montréal/CRCHUM, 900, rue St-Denis, porte R04-446, Montreal, Québec H2X 0A9 Canada

**Keywords:** Apalutamide, Castration-resistant prostate cancer, Ventricular repolarization, QT interval, Pharmacokinetics

## Abstract

**Purpose:**

Phase Ib study evaluating the effect of apalutamide, at therapeutic exposure, on ventricular repolarization by applying time-matched pharmacokinetics and electrocardiography (ECG) in patients with castration-resistant prostate cancer. Safety of daily apalutamide was also assessed.

**Methods:**

Patients received 240 mg oral apalutamide daily. Time-matched ECGs were collected via continuous 12-lead Holter recording before apalutamide (Day − 1) and on Days 1 and 57 (Cycle 3 Day 1). Pharmacokinetics of apalutamide were assessed on Days 1 and 57 at matched time points of ECG collection. QT interval was corrected for heart rate using Fridericia correction (QTcF). The primary endpoint was the maximum mean change in QTcF (ΔQTcF) from baseline to Cycle 3 Day 1 (steady state). Secondary endpoints were the effect of apalutamide on other ECG parameters, pharmacokinetics of apalutamide and its active metabolite, relationship between plasma concentrations of apalutamide and QTcF, and safety.

**Results:**

Forty-five men were enrolled; 82% received treatment for ≥ 3 months. At steady state, the maximum ΔQTcF was 12.4 ms and the upper bound of its associated 90% CI was 16.0 ms. No clinically meaningful effects of apalutamide were reported for heart rate or other ECG parameters. A concentration-dependent increase in QTcF was observed for apalutamide. Most adverse events (AEs) (73%) were grade 1–2 in severity. No patients discontinued due to QTc prolongation or AEs.

**Conclusion:**

The effect of apalutamide on QTc prolongation was modest and does not produce a clinically meaningful effect on ventricular repolarization. The AE profile was consistent with other studies of apalutamide.

## Introduction

Prostate cancer is the second most common cancer in men worldwide, accounting for 15% of cancers diagnosed in men [1]. Metastatic castration-resistant prostate cancer (mCRPC) is associated with progressive morbidities, including skeletal-related events [2]. Because prostate cancer cells depend on the androgen receptor (AR) for survival and growth, treatment for recurrent or primary metastatic prostate cancer targets this receptor axis [3]. Despite initial therapies that target the AR, many patients progress to CRPC [3]. Apalutamide is an orally administered next-generation AR inhibitor currently approved in the United States for patients with nonmetastatic CRPC (nmCRPC) [4]. It directly binds the ligand-binding domain of the AR, inhibits AR nuclear translocation and DNA binding, and impedes AR-mediated transcription [5].

The efficacy and safety of apalutamide were demonstrated in patients with prostate cancer in the SPARTAN study, a randomized, double-blind, placebo-controlled, multicenter trial that evaluated apalutamide treatment in 1207 patients with high-risk nmCRPC [6]. This study was the first to demonstrate a significantly longer median metastasis-free survival (MFS; 2 years over placebo) in apalutamide-treated patients compared with placebo-treated patients, with consistent benefit for apalutamide across all secondary endpoints, including time to symptomatic progression [6]. Minimal cardiac adverse events (AEs) were observed; atrial fibrillation was cited as the primary cardiac-associated AE reason for dose interruption and occurred in 0.7 and 0.5% of patients in the apalutamide and placebo arms, respectively. Based on these data, apalutamide was approved in February 2018 by the US Food and Drug Administration (FDA) for the treatment of men with nmCRPC [4].

Apalutamide pharmacokinetics (PK) has been well characterized in clinical studies. Apalutamide is rapidly absorbed, with a median time to maximum observed plasma concentration (*C*_max_) of 2–3 h after oral administration [7]. Additionally, PK was approximately proportional across dose levels, with a mean effective half-life of approximately 3 days after multiple doses (Data on file, Janssen). Steady state exposure was achieved following 4 weeks of continuous, daily apalutamide administration [4, 7]. For *N*-desmethyl apalutamide, a minimal peak to trough fluctuation ratio in plasma at steady state (≈ 1.3) was observed. Time to reach *C*_max_ (*t*_max_) for *N*-desmethyl apalutamide at steady state was variable and typically occurred at around 1 or 24 h post dose (Data on file, Janssen).

The preclinical cardiovascular safety of apalutamide and its active metabolite *N*-desmethyl apalutamide has been assessed in in vitro and in vivo studies (Data on file, Janssen). Both apalutamide and *N*-desmethyl apalutamide inhibited human ether-à-go-go-related (hERG) gene current, with half maximal inhibitory concentration (IC_50_) values of 6.17 and 4.56 µM, respectively, representing a safety margin of at least seven relative to the anticipated *C*_max_ for unbound apalutamide and *N*-desmethyl apalutamide in patients at the clinical dose of 240 mg/day (Data on file, Janssen). No relevant effect was produced (no prolongation in action potential duration; no effect on resting membrane potential) in isolated canine Purkinje fibers with up to 30 µM of apalutamide or *N*-desmethyl apalutamide (Data on file, Janssen). Preclinical in vitro receptor binding assay testing did not reveal an effect of apalutamide on Na^+^ or Ca^2+^ channels (Data on file, Janssen). No in vivo treatment-related cardiovascular effects (blood pressure, heart rate, body temperature, ECG lead II intervals, PR, QRS, QT, QTc, or ECG waveform morphology) were noted in a single-dose telemetry study in conscious dogs up to 40 mg/kg or after repeated dosing in good laboratory practice toxicology studies in dogs up to 10 mg/kg/day with exposures in the range of the clinical exposure for apalutamide and its metabolite *N*-desmethyl apalutamide (Data on file, Janssen). Overall preclinical cardiovascular safety assessment of apalutamide was not indicative of an increased risk for QTc prolongation in clinical use (Data on file, Janssen).

The effect of apalutamide on ventricular repolarization was previously evaluated as part of a phase I/II study [7] that included time-matched triplicate 12-lead electrocardiograms (ECGs) collected at baseline and at steady state in 12 patients with CRPC. The data showed no significant effect from apalutamide on ECG parameters, and there was no conclusive evidence for an increase in Fridericia corrected QT interval (QTcF) (Data on file, Janssen).

Androgen deprivation therapy (ADT) can increase cardiovascular risk because of its adverse effect on risk factors for cardiovascular disease [8, 9]. Combination treatment with bicalutamide plus LHRH agonist therapy and 150-mg bicalutamide monotherapy may lead to QTc prolongation [10–12]. In AFFIRM, a randomized phase III study, the effect of enzalutamide (160 mg/day) on QTcF was assessed at steady state in 800 patients with CRPC [13]. No clinically meaningful changes were observed between the mean QTcF interval change from baseline in patients treated with enzalutamide versus those treated with placebo [13]. A recent post hoc analysis of the TERRAIN study suggests a higher risk for atrial fibrillation in patients with mCRPC taking enzalutamide (160 mg/day) versus bicalutamide (50 mg/day) [14].

Because drug-induced QT interval prolongation has been one of the most common causes of drug withdrawals or restrictions of already marketed drugs [15, 16], a thorough premarketing assessment of a drug’s potential to cause ECG change or generate life-threatening arrhythmias is a regulatory requirement detailed in the International Conference on Harmonisation of Technical Requirements for Registration of Pharmaceuticals for Human Use—Harmonised Tripartite Guideline E14 (ICH E14) [17]. Therefore, the current study evaluated the effect of therapeutic doses of apalutamide (240 mg) and its active metabolite, *N*-desmethyl apalutamide, on ventricular repolarization in patients with CRPC.

In accordance with the ICH E14 guideline, a thorough QT (TQT) study ideally has a four-way crossover design, including a therapeutic dose, a supratherapeutic dose, a placebo, and a positive control. In light of the absence of a preclinical QT signal and no conclusive evidence for QTc prolongation in a previous phase I/II study, combined with the need for ≥ 8 weeks of dosing with apalutamide to reach steady state conditions, providing 8 weeks of placebo treatment in a cancer population would be unethical. The implementation of a positive control would have required the standalone administration of a positive control (e.g., moxifloxacin) and adequate washout prior to starting apalutamide treatment, to avoid any carryover effect on the predose (Day − 1) and Cycle 1 Day 1 (C1D1) ECG assessments. Moreover, there is limited safety experience with apalutamide at a dose of > 240 mg from previous clinical studies. Thus, an alternative multiple dose-dedicated QTc study design was chosen and customized for the oncological setting and for the PK characteristics of apalutamide.

## Materials and methods

### Patients

Enrolled patients were diagnosed with adenocarcinoma of the prostate and with either high-risk nmCRPC [prostate-specific antigen (PSA) doubling time ≤ 10 months] or mCRPC. Other inclusion factors were surgical or medical castration with testosterone levels < 50 ng/dL, Eastern Cooperative Oncology Group (ECOG) performance status 0 or 1, adequate bone marrow and organ function, QTcF ≤ 470 ms, and left ventricular ejection fraction of > 45%. Key exclusion criteria included known brain metastases, prior treatment with enzalutamide or apalutamide, grade ≥ 2 electrolyte abnormalities (hypokalemia, hypocalcemia, hypomagnesemia), uncontrolled hypertension, significant cardiac function abnormalities on screening ECG, and history or evidence of certain cardiac conditions. Patients requiring concurrent therapy with medications known to be associated with QTc interval prolongation and an increased risk of torsades de pointes were excluded from the study. Per protocol, strong CYP3A4 inducers, strong CYP2C8 inducers (e.g., rifampin), and strong CYP2C8 inhibitors (e.g., gemfibrozil) were prohibited to not influence apalutamide exposure levels.

### Study design

This was an open-label, multicenter, phase Ib study to investigate the effect of daily apalutamide (240 mg, orally) on ventricular repolarization (QTc). Approximately 42 patients with high-risk nmCRPC (defined as having a PSA doubling time of ≤ 10 months) or mCRPC were planned to be enrolled to ensure that at least 38 patients completed the study.

The study consisted of screening, treatment, and follow-up phases. After providing informed consent, the patients entered a 28-day screening phase for determination of eligibility. If eligible, patients began the open-label treatment phase and were monitored for PK, pharmacodynamics [(PD) ECGs], and safety (including cardiac safety). Apalutamide was administered in continuous 28-day treatment cycles. The duration of the treatment phase for PD (ECG) and PK data collection was from baseline on C1D−1 until C3D1 (Day 57). Patients were allowed to continue apalutamide treatment after C3D1 until disease progression, withdrawal of consent, loss to follow-up, the occurrence of unacceptable toxicity, or loss of clinical benefit (investigator opinion). The follow-up phase for AEs lasted from discontinuation of apalutamide until 30 days after the last dose. Upon discontinuation of study drug, patients returned once for an end-of-treatment (EoT) visit ≤ 30 days after their last dose.

### Pharmacodynamic (ECG) evaluations

Patients were admitted to the study center on C1D−1 (baseline), C1D1, and C1D3 for PK/PD evaluation. Study drug intake was planned at 9:00 a.m. on C1D1 and C1D3 after overnight fasting. Continuous 12-lead ECGs were collected by a Holter monitor on C1D−1, C1D1, and C3D1 from 8:00 a.m. to 3:00 p.m. Triplicate 12-lead ECGs were obtained during a 10-min time interval at the following time points: at predose (− 0.5 h) and at 1, 2, 3, 4, and 5 h after apalutamide administration. This 10-min timeframe began 5 min before and ended 5 min after each scheduled time point. Holter recordings were sent to a blinded, third-party, central ECG contract laboratory for 12-lead ECG selection/extraction, ECG interval measurements, and ECG interpretation.

ECG parameters measured included QRS (the onset of ventricular depolarization), PR (the period extending from the beginning of the onset of atrial depolarization until the beginning of the QRS complex), RR (with R being the point corresponding to the peak of the QRS complex of the ECG wave, and RR being the interval between successive Rs), and QT (electrical depolarization and repolarization of the ventricles).

### Pharmacokinetic evaluations

After apalutamide administration on C1D1 and C3D1, time-matched PK blood samples were collected ≤ 5 min after completion of the 10-min timeframe for planned ECG collection at predose (− 0.5 h) and at 1, 2, 3, 4, and 5 h postdose. To calculate an area under the curve (AUC) value over 24 h after the first dose (C1D1), a 24-h PK sample was also collected during C1D2. Plasma concentrations of apalutamide and *N*-desmethyl apalutamide were determined using validated liquid chromatography/tandem mass spectrometry methods. The assay consisted of protein precipitation followed by liquid chromatography with tandem mass spectrometric detection. Stable isotope-labeled internal standards were used for quantification. Chromatography was performed with a Waters Xbridge C18 column (50 × 2.1 mm, 3.5 µm) using a gradient with 0.1% formic acid and acetonitrile. An API5000 mass spectrometer in the negative ion mode with a Turbo-Ionspray Interface (AB SCIEX, MA, USA) was used. Multiple reaction monitoring (MRM) transitions were from *m/z* 476.1 to 419.1 and from 479.1 to 419.1 for apalutamide and the internal standard, respectively. For *N*-desmethyl apalutamide and the internal standard, respectively, MRM transitions were from *m/z* 464.1 to 235.0 and from 469.2 to 240.1. The quantification range was 0.0250–25.0 µg/mL for both analytes, and the assay performance was monitored using quality control samples. The recorded values all met the acceptance criteria.

PK parameters calculated for apalutamide and *N*-desmethyl apalutamide using noncompartmental analysis included *C*_max_, *t*_max_, AUC from time 0 to 24 h after dosing (AUC_24h_), and the minimum observed plasma concentration (*C*_min_). Additionally, the accumulation index (AI), metabolite:parent drug ratio, corrected for molecular weight (MPR; for *N*-desmethyl apalutamide only), and peak/trough ratio at steady state (PTR) were also calculated.

The PK/PD data collection sought to determine the potential relationship between change from baseline in QTc (ΔQTc) and the plasma concentrations of apalutamide and *N*-desmethyl apalutamide. The measured QT data were corrected for heart rate using Fridericia (QTcF) [18], Bazett (QTcB), and a study-specific correction Power (QTcP) correction method. The correlation between QTcF and RR was not significant, with a slope of 0.031 and a 90% confidence interval (CI) (− 0.01 to 0.07) that included zero. A similar analysis with the QTcP method also showed no statistically significant relationship between QTc and RR, whereas with the QTcB method a statistically significant relationship between QTc and RR was observed. Overall, these analyses support the use of the QTcF method as the primary correction method; thus, only QTcF data are reported herein. Baseline was defined as the mean QTc values of the triplicate ECG measurements taken at baseline. These baseline QTc values were time-matched with those on C1D1 and C3D1. The ΔQTc was calculated at each time point. The primary endpoint was the maximum mean ΔQTc on C3D1, which was estimated by the mean ΔQTc at around *t*_max_ (i.e., steady state). The duration of PD and PK assessments during the treatment phase was from baseline until C3D1.

### Safety evaluations

Patients were monitored for safety during the screening, treatment, and follow-up phases until 30 days after the last dose of study drug. The safety evaluations included AE reporting, clinical laboratory safety evaluations, ECGs, multigated acquisition scan, or echocardiogram (screening only for determination of left ventricular ejection fraction), ECOG performance status scores, vital signs, and physical examination. For patients who remained in the study after three cycles of apalutamide treatment, collection of AEs was limited to serious AEs (SAEs) and grade ≥ 3 AEs. Patients were followed for disease progression as clinically indicated per institutional guidelines, which could include PSA monitoring or imaging collected at the discretion of the investigator. The safety population included all patients who received at least one dose of apalutamide.

### Statistical analysis

The clinical cutoff for the statistical analysis of the study was defined when the last patient enrolled had completed the C3D1 (Day 57) assessments. For the statistical analysis based on the primary correction method (QTcF), the mean changes from baseline (ΔQTcF) at each time point were summarized [mean, standard deviation (SD), median and range, two-sided 90% CI]. The primary endpoint analysis focused on the maximum mean ΔQTcF at C3D1, which was estimated by the mean QTcF change at around *t*_max_, the time when *C*_max_ was reached. The mean ΔQTcF (± SD) over time was plotted. For each treatment and time point of measurement, HR, QRS, PR, and RR intervals, as well as the change from baseline in HR, QRS, PR, and RR (ΔHR, ΔQRS, ΔPR, ΔRR), were summarized using descriptive statistics (mean, SD, median, range, and 90% CI).

Individual plasma concentrations for apalutamide and its active metabolite *N*-desmethyl apalutamide were tabulated with descriptive statistics (including arithmetic mean, SD, coefficient of variation, median, minimum, and maximum) at each sampling time point for each visit. Individual and mean plasma concentration–time profiles were plotted.

A linear mixed-effects model was fitted to the ΔQTc data from C1D1 and C3D1 with either parent or metabolite concentration as a predictor and patient as a random effect. On the basis of these relationships, the predicted population average ΔQTc and its corresponding upper 90% two-sided CI bound were computed at the mean *C*_max_ of apalutamide and *N*-desmethyl apalutamide, or other concentrations of interest.

## Results

### Patient and disease characteristics

At the time of clinical cutoff, 45 men enrolled at five study centers received at least one dose of apalutamide and were included in the safety analysis set. At study entry, the majority (97.8%) of patients had mCRPC. One patient had high-risk nmCRPC. Forty three of the 45 patients were considered study completers (defined as having completed C3D1 ECG collection and PK sampling procedures in the presence of adequate compliance with intake of the study drug during Cycles 1 and 2) and were included in the primary analysis set. Median age at study entry was 67 years (range 52–86 years) (Table 1). All patients had received therapy for prostate cancer prior to study entry in addition to ADT or surgical castration; the most commonly prescribed prior therapies were bicalutamide (89%), abiraterone acetate (42%), and docetaxel (38%) (Table 1). Overall, study participants were largely compliant with avoidance of prohibited medications that could influence apalutamide or *N*-desmethyl apalutamide PK.

**Table 1:** Patient and disease characteristics

Baseline characteristic	Total (*N* = 45)
Median age, year (range)	67 (52–86)
Race, *n* (%)	
White	42 (93)
Black or African American	3 (7)
Median weight, kg (range)	80 (50–135)
Median time from initial diagnosis, mo (range)	68.2 (3.9–280.3)
Extent of disease, *n* (%)	
Bone	40 (91)
Soft tissue or node	14 (32)
Liver	2 (4)
Lung	1 (2)
Other	3 (7)
None	1 (2)
ECOG PS, *n* (%)	
0	27 (60)
1	18 (40)
Median testosterone, nM (range)	0.85 (0.07–1.63)
Prior therapy, *n* (%)	45 (100)
Bicalutamide	40 (89)
Abiraterone acetate	19 (42)
Docetaxel	17 (38)
Cabazitaxel	11 (24)

*ECOG PS* Eastern Cooperative Oncology Group performance status

### Patient disposition

At the time of clinical cutoff, the median treatment duration was 5 months (range 2–8 months); 82% of patients had received treatment for ≥ 3 months. Furthermore, 13 of 45 patients had discontinued treatment. Treatment was discontinued due to progressive disease in 12 patients, and one patient withdrew consent.

### Primary endpoint

A total of 831 evaluable ECGs were reviewed in this study, out of 855 expected ECG extractions. For each QTc correction method, the relation between QTc and RR at baseline was evaluated graphically by plotting the logarithm of baseline QTc values against the logarithm of the corresponding RR intervals. This analysis supported the QTcF method being used as the primary correction method. The primary endpoint was maximum mean ΔQTcF on C3D1. There was no notable difference in QTcF intervals between baseline (Day − 1) and after the first dosing (C1D1). For all the corresponding time points on C3D1, there were mean increases from baseline in QTcF, but no obvious time-related trends over the course of the day. The least squares mean increases from baseline on C3D1 ranged from 8.0 to 12.4 ms (Table 2). The least squares mean (standard error) QTcF change at *t*_max_ on C1D1 and on C3D1 was + 1.9 (1.6) ms and + 12.4 (2.1) ms, respectively. The upper limit of the 90% CI of the least squares mean baseline corrected QTcF change at each postdose time point was below 10 ms for C1D1 (maximum of upper limits = 4.5 ms) and above 10 ms, for C3D1 (maximum of upper limits = 16.0 ms).

**Table 2:** Mean ΔQTcF over time after single dose and steady state

	Absolute QTcF interval^a^, ms			ΔQTcF (LS mean) interval^b^, ms		
	*N*	Mean (SD)	95% CI	*N*	Mean (SE)	90% CI
C1D1						
Predose	43	428.7 (13.5)	(424.5–432.9)	43	− 0.7 (1.59)	− 3.4 to 1.9
1 h	42	430.1 (14.5)	(425.6–434.6)	41	− 0.4 (1.62)	− 3.1 to 2.3
2 h	43	432.4 (15.2)	(427.7–437.0)	42	1.9 (1.61)	− 0.8 to 4.5
3 h	43	424.7 (13.9)	(420.4–429.0)	42	− 3.1 (1.61)	− 5.8 to − 0.4
4 h	43	425.7 (14.5)	(421.3–430.2)	42	− 2.1 (1.61)	− 4.8 to 0.6
5 h	43	422.4 (15.9)	(417.5–427.3)	41	− 5.5 (1.62)	− 8.2 to − 2.8
C3D1						
Predose	42	441.6 (16.8)	(436.3–446.8)	42	12.0 (2.14)	8.4–15.5
1 h	42	442.7 (18.6)	(436.9–448.5)	41	12.3 (2.16)	8.7–15.9
2 h	43	442.9 (16.4)	(437.8–447.9)	42	12.4 (2.15)	8.8–16.0
3 h	42	439.3 (15.7)	(434.4–444.2)	41	10.9 (2.15)	7.3–14.5
4 h	42	436.5 (14.2)	(432.0–440.9)	41	8.2 (2.15)	4.6–11.8
5 h	42	436.0 (16.3)	(430.9–441.1)	40	8.0 (2.16)	4.4–11.6

*CI* confidence interval, *LS* least squares, *SD* standard deviation, *SE* standard error, *C1D−1* Cycle 1 Day − 1, *C1D1* Cycle 1 Day 1, *C3D1* Cycle 3 Day 1

^a^The time-matched baseline is defined as the mean values of the triplicate electrocardiographic measurements taken on C1D−1, at the time points matching with those on C1D1 and C3D1

^b^A repeated-measures mixed model was used with time point and baseline value of QTc as fixed effect, and patient as a random effect

Patients with QTcF intervals exceeding the threshold values of 450, 480, and 500 ms are summarized in Table 3. Twelve patients had QTcF value > 450 and ≤ 480 ms at baseline while the number increased to 20 patients on C3D1. One patient had a QTcF interval > 480 and ≤ 500 ms at 1 h postdose on C3D1; this same patient also had a predose (C1D1, at − 1 h) QTcF value of 469.3 ms. No QTcF intervals > 500 ms were recorded. Numbers of patients with a QTcF interval change from baseline exceeding the threshold values of 30 or 60 ms are also summarized in Table 3. Two patients on C1D1 and nine patients on C3D1 had a QTcF interval change from baseline of > 30 but ≤ 60 ms. Among the patients with QTcF interval changes from baseline of > 30 ms but ≤ 60 ms, no association was observed with the presence of underlying electrolyte abnormalities or significant cardiac medical history. One patient had a QTcF interval change from baseline of 60.4 ms at 1 h postdose on C3D1 (455.7 ms). The two patients with QTcF > 480 ms or with QTcF increase of > 60 ms from baseline did not have a significant cardiac medical history and did not use any concomitant medications with a liability for QTc prolongation. The patient with QTcF > 480 ms on C3D1 showed grade 1 hypocalcemia at baseline and C3D1, but potassium and magnesium levels were normal.

**Table 3:** Categorical analysis of QTcF at baseline and post apalutamide treatment

	Baseline (*N* = 43)	C1D1 (*N* = 43)	C3D1 (*N* = 43)	Total (C1D1 + C3D1) (*N* = 43)
Pre- or post-apalutamide QTcF > 450 ms, *n* (%)^a^				
> 450 to ≤ 480 ms	12 (28)	6 (14)	20 (47)	20 (47)
> 480 to ≤ 500 ms	0	0	1 (2)	1 (2)
> 500 ms	0	0	0	0
QTcF increase from baseline > 30 ms, *n* (%)^b^				
> 30 to ≤ 60 ms	–	2 (5)	9 (21)	9 (21)
> 60 ms	–	0	1 (2)	1 (2)

*C1D−1* Cycle 1 Day − 1, *C1D1* Cycle 1 Day, *C3D1* Cycle 3 Day 1

^a^Percentages are calculated with the number of patients in primary analysis as denominators and only the worst value for a patient is presented; the C1D1 predose measurement and C1D−1 measurement are considered as baseline

^b^Percentages are calculated with the number of patients in primary analysis as denominators; time-matched baseline is defined as the mean values of the triplicate electrocardiographic measurements taken on C1D−1 (including predose), at the time points matching with those on C1D1 (including predose) and C3D1 (including predose)

### Secondary endpoints

For all time points on C1D1, mean heart rate was increased from baseline (Table 4), without obvious time-related trends over the course of the day. The mean increases from baseline on C1D1 ranged from 0.1 to 2.5 bpm. For all time points on C3D1, there were mean decreases from baseline in HR, but with no obvious time-related trends over the course of the day. The mean decreases from baseline on C3D1 ranged from − 0.4 to − 3.5 bpm. The number of patients with any HR [> 100 bpm (*n* = 3) or < 50 bpm (*n* = 2)] was similar at baseline and on C1D1 and C3D1 (data not shown). Apalutamide did not have a clinically significant effect on HR.

**Table 4:** Change from baseline in heart rate, RR interval, PR interval, and QRS interval at C1D1 and C3D1

	*N*	Mean ± SD	90% CI
Heart rate, bpm			
C1D1			
Predose	43	0.6 ± 5.83	− 0.9 to 2.1
1 h	41	0.7 ± 5.96	− 0.9 to 2.3
2 h	42	2.4 ± 13.11	− 1.0 to 5.8
3 h	42	2.5 ± 11.97	− 0.6 to 5.7
4 h	42	0.1 ± 13.26	− 3.4 to 3.5
5 h	41	2.2 ± 6.01	0.6 to 3.8
C3D1			
Predose	42	− 2.5 ± 6.09	− 4.0 to − 0.9
1 h	41	− 1.7 ± 5.93	− 3.2 to − 0.1
2 h	42	− 3.4 ± 6.60	− 5.1 to − 1.7
3 h	41	− 1.3 ± 13.69	− 4.9 to 2.3
4 h	41	− 3.5 ± 14.82	− 7.4 to 0.4
5 h	40	− 0.4 ± 7.17	− 2.3 to 1.5
RR interval, ms			
C1D1			
Predose	43	− 7.5 ± 89.97	− 30.6 to 15.5
1 h	41	− 13.3 ± 92.29	− 37.6 to 11.0
2 h	42	− 16.1 ± 92.55	− 40.1 to 8.0
3 h	42	− 16.0 ± 83.55	− 37.7 to 5.7
4 h	42	− 6.4 ± 86.57	− 28.9 to 16.1
5 h	41	− 21.5 ± 63.13	− 38.1 to 4.9
C3D1			
Predose	42	39.0 ± 89.10	15.8 to 62.1
1 h	41	27.5 ± 84.93	5.2 to 49.9
2 h	42	41.0 ± 77.88	20.8 to 61.2
3 h	41	33.2 ± 143.13	− 4.5 to 70.8
4 h	41	36.4 ± 141.19	− 0.7 to 73.5
5 h	40	6.3 ± 79.87	− 15.0 to 27.6
PR interval, ms			
C1D1			
Predose	43	2.6 ± 12.34	− 0.5 to 5.8
1 h	41	1.9 ± 13.01	− 1.6 to 5.3
2 h	41	− 0.7 ± 10.12	− 3.4 to 2.0
3 h	41	− 1.1 ± 9.78	− 3.7 to 1.5
4 h	41	− 1.8 ± 9.51	− 4.3 to 0.7
5 h	41	− 0.3 ± 6.31	− 2.0 to 1.4
C3D1			
Predose	42	2.2 ± 13.70	− 1.3 to 5.8
1 h	41	0.1 ± 17.29	− 4.4 to 4.7
2 h	42	1.6 ± 11.38	− 1.4 to 4.5
3 h	40	− 0.9 ± 15.72	− 5.1 to 3.3
4 h	40	− 2.7 ± 14.82	− 6.6 to 1.3
5 h	40	0.6 ± 8.54	− 1.7 to 2.8
QRS interval, ms			
C1D1			
Predose	43	0.5 ± 3.36	− 0.4 to 1.4
1 h	41	0.9 ± 3.35	0.0 to 1.8
2 h	42	0.5 ± 3.97	− 0.5 to 1.6
3 h	42	0.8 ± 3.58	− 0.2 to 1.7
4 h	42	0.9 ± 3.00	0.1 to 1.6
5 h	41	0.5 ± 3.53	− 0.4 to 1.5
C3D1			
Predose	42	2.4 ± 4.53	1.2–3.6
1 h	41	1.6 ± 5.05	0.3–2.9
2 h	42	1.9 ± 4.91	0.7–3.2
3 h	41	2.2 ± 4.88	0.9–3.4
4 h	41	1.6 ± 5.07	0.3–3.0
5 h	40	2.3 ± 4.71	1.1–3.6

*SD* standard deviation, *CI* confidence interval, *C1D−1* Cycle 1 Day − 1, *C1D1* Cycle 1 Day 1, *C3D1* Cycle 3 Day 1

For all time points on C1D1, there were mean decreases from baseline in RR interval but with no obvious time-related trends over the course of the day (Table 4). The decreases from baseline on C1D1 ranged from − 6.4 to − 21.5 ms. For all time points on C3D1, there were mean increases from baseline in RR interval, also with no obvious time-related trends over the course of the day. The increases from baseline on C3D1 ranged from 6.3 to 41.0 ms. The observations on the RR interval are inversely correlated with the observations on HR interval.

For mean PR interval over time compared with baseline, no obvious time-related trends were noted over the course of C1D1 or C3D1 (Table 4). The incidence count and percentage of patients with any PR interval > 200 ms by study day and by time point was similar at baseline and on C1D1 or C3D1 (data not shown). No effect of apalutamide on the length of the PR interval was apparent. For all time points on C1D1 and C3D1, mean increases were observed from baseline in the QRS interval (Table 4). The mean increases from baseline on C1D1 ranged from 0.5 to 0.9 ms and on C3D1 from 1.6 to 2.4 ms. No patients had a QRS interval > 110 ms at baseline or on C1D1. QRS intervals > 110 ms but ≤ 115 ms on C3D1 were recorded in three patients. The largest mean change (increase) in QRS duration from baseline was 2.4 ms on C3D1 at predose. Overall, no clinically relevant effects of apalutamide on the QRS interval were observed.

T-wave morphology was monitored, and the number of patients with flat, inverted, or biphasic T-waves was similar on pretreatment and post-treatment days. For most patients with T-wave abnormalities observed during the treatment phase, these observations were also noted on predose ECG before apalutamide administration. De novo T-wave abnormalities were observed in three patients (7%), which were absent at baseline, and no QTcF prolongation ≥ 480 ms was observed in these three patients. Apalutamide treatment did not have an apparent association with the appearance or worsening of T-wave abnormalities, and no U-waves were observed in any patient.

**Fig. 1:**
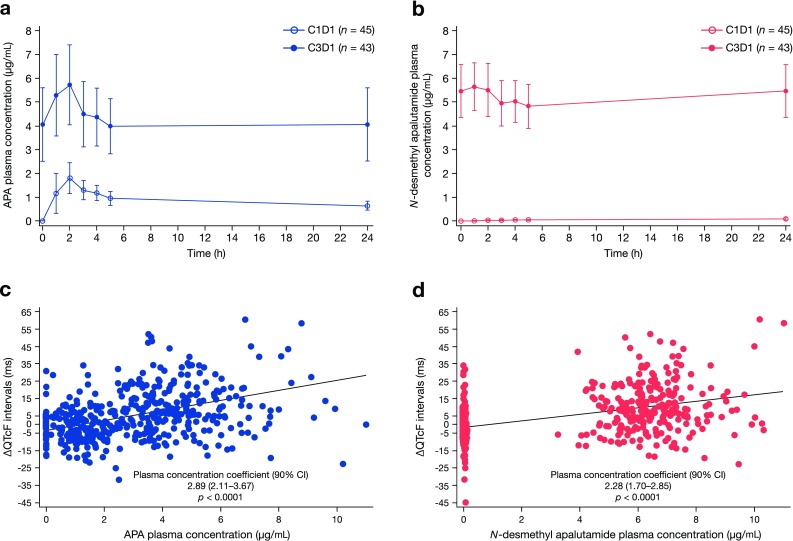
Plasma concentration of apalutamide and *N*-desmethyl apalutamide and their association with QTcF: **a** mean plasma concentration–time profiles of apalutamide after administration of 240 mg apalutamide on C1D1 and C3D1; **b** mean plasma concentration–time profiles of *N*-desmethyl apalutamide after administration of 240 mg apalutamide on C1D1 and C3D1; **c** scatter plot of the relationship between ΔQTcF and plasma concentration of apalutamide; **d** scatter plot of the relationship between ΔQTcF and plasma concentration of *N*-desmethyl apalutamide

Mean plasma concentrations over time for apalutamide and *N*-desmethyl apalutamide are shown in Fig. 1a, b. Repeated once-daily administration of 240-mg apalutamide under fasted conditions resulted in a three- and fivefold increase of *C*_max_ and AUC_24h_, respectively, when comparing apalutamide systemic exposure between C1D1 and C3D1 (Table 5). Median *t*_max_ was reached at approximately 2 h post dose on C1D1 and C3D1. At steady state (C3D1), the active metabolite N-desmethyl apalutamide exhibited a flat PK profile with a mean PTR of 127%. The MPRs for *C*_max_ and AUC_24h_ were 105 ± 21 and 133 ± 28%, respectively (Table 5). A significant correlation was observed between the change in QTcF from baseline and the concentration of apalutamide (slope estimate, 2.89; 90% CI, 2.11–3.67; *p* < 0.0001) (Fig. 1c). The predicted ΔQTcF (90% CI) at a mean *C*_max_ of 5.95 µg/mL was 13.8 ms (9.77–17.85). Likewise, a significant correlation was observed between the change in QTcF from baseline and the concentration of *N*-desmethyl apalutamide (slope estimate, 2.28; 90% CI 1.70–2.85; *p* < 0.0001) (Fig. 1d). For instance, on C3D1 the predicted ΔQTcF (90% CI) at a mean steady state *C*_max_ of 5.84 µg/mL was 12.0 ms (8.58–15.38).

**Table 5:** Pharmacokinetics of apalutamide and *N*-desmethyl apalutamide

Parameter^a^	Apalutamide		*N*-desmethyl apalutamide	
	C1D1 (*N* = 45)	C3D1 (*N* = 43)	C1D1 (*N* = 45)	C3D1 (*N* = 43)
*C*_max_, µg/mL	2.06 (0.58)	5.95 (1.66)	0.092 (0.057)	5.85 (1.04)
*t*_max_, h	2.12 (1.08–5.10)	2.10 (1.00–4.17)	24.00 (4.10–24.58)	1.10 (0.00–4.17)
AUC_24h_, µg·h/mL	21.1 (4.93)	100 (31.6)	1.41 (0.79)	124 (23.0)
*C*_min_, µg/mL	–	3.72 (1.19)	–	4.66 (0.90)
PTR, %	–	163 (24.7)	–	127 (13.3)
AI_(*C*max)_	–	3.09 (1.26)	–	82.1 (50.5)
AI_(AUC24h)_	–	4.95 (1.69)	–	122 (108)
MPR *C*_max_, % (SD)	–	–	–	105 (20.8)
MPR AUC_24h_, % (SD)	–	–	–	133 (28.0)

*C1D1* Cycle 1 Day 1, *C3D1* Cycle 3 Day 1, *C*_*max*_ maximum observed plasma concentration, *t*_*max*_ time to *C*_max_, *AUC*_*24h*_ AUC from time 0 to 24 h after dosing, *C*_*min*_ minimum observed plasma concentration, *PTR* peak/trough ratio at steady state, *AI* accumulation index, *MPR* metabolite:parent drug ratio corrected for molecular weight

^a^All values are presented as the mean (SD) except for *t*_max_, which is presented as the median (range), or as otherwise noted

### Safety

Dose modifications were allowed for toxicity attributed to apalutamide, and re-escalation was permitted if first discussed with and approved by the sponsor. The majority of patients had neither dose reduction (42 patients, 93%) nor dose interruption (38 patients, 84%).There were no dose re-escalations after initial dose reduction. Drug-related toxicities leading to temporary dose interruption included grade 3 diarrhea and aspartate transaminase/alanine transaminase increase and grade 2 fatigue. Two patients required a dose reduction due to fatigue. Thirteen (29%) patients discontinued treatment, with 12 of those discontinuing due to progressive disease and one due to withdrawal of consent (no discontinuations were due to AEs).

Thirty-seven (82%) patients experienced at least one treatment-emergent AE (TEAE), most of which were grades 1–2. The most commonly reported TEAEs (≥ 10% of patients) were fatigue (40%), decreased appetite (24%), back pain (16%), diarrhea and dyspnea (13% each), rash/erythema (13%), and constipation and nausea (11% each). No treatment-emergent seizures were reported. The AEs recorded in this study were consistent with those observed in other published apalutamide studies [6, 7, 19, 20]. Grade 3 TEAEs were reported in eight (18%) patients and grade 4 AEs in two (4%) patients. Grade 3 TEAEs reported in > 1 patient were anemia (3 patients, 7%) and back pain (2 patients, 4%); these grade 3 TEAEs were not considered related to apalutamide treatment. Three patients reported grade 3 toxicities considered possibly or probably related to apalutamide, including fatigue, diarrhea, and aspartate transaminase/alanine transaminase increase. In one patient, grade 3 cardiac failure was reported on study Day 45 and was not considered related to apalutamide treatment. Grade 3 nervous system disorder and spinal cord compression were reported in two patients, were not considered related to apalutamide treatment, and occurred in an overall context of worsening vertebral metastatic disease. One patient experienced grade 4 thrombocytopenia, which was not considered related to apalutamide treatment and occurred in the context of progressive disease. Another patient had grade 4 neutropenia not considered related to apalutamide treatment that occurred while the patient took sulfamethoxazole plus trimethoprim for a bladder infection.

Five patients experienced ≥ 1 SAE, but none were considered related to apalutamide treatment; SAEs were grades 1–3, except for a grade 4 SAE of general health deterioration in one patient, who subsequently died from progressive disease. One patient experienced a grade 3 SAE of medullary compression, which was considered not related to apalutamide treatment and likely related to underlying metastatic disease in the vertebra. Another patient with bone metastases experienced a grade 3 SAE of progressive lower back pain, which was attributed to the magnetic resonance imaging-verified metastatic disease in the pelvis. One patient experienced multiple SAEs: grade 2 hypercalcemia, grade 3 jaw necrosis, grade 3 pain left hip, and grade 3 neurologic deficit due to spinal cord compression resulting from bone metastases; none were considered related to apalutamide treatment. The final patient, a 70-year-old man with a history of hypertension, mitral valve prolapse, and type 2 diabetes, experienced multiple SAEs, including grade 3 lower back pain, grade 2 infection, and grade 2 delirium, none of which were considered related to apalutamide treatment. This patient also experienced a grade 3 SAE of heart failure caused by de novo atrial fibrillation that was not considered related to apalutamide treatment.

Laboratory values were collected over time (data not shown). Most patients had occasional changes in serum chemistry and some hematologic abnormalities, the majority of which were grade 0–2 in severity. Elevated thyroid-stimulating hormone (TSH) levels above the upper limit of normal during the study were observed in 13 patients (29%), were usually limited in magnitude and, in the majority of cases, thyroid hormone levels stayed within normal limits. In two patients (4%), significant TSH elevations were observed in combination with a decrease in thyroid hormone levels. One of these two patients had a medical history of hypothyroidism and required an increase in thyroid supplementation therapy in the course of the study.

## Discussion

These data from an open-label, multicenter, phase Ib dedicated QT/QTc study that investigated the effect of apalutamide on ventricular repolarization and other ECG parameters confirm the absence of major effects from apalutamide on the QTc interval in men with CRPC. This modified QTc study, tailored to the oncologic setting and taking into account the PK characteristics of apalutamide, was rigorously executed with time-matched ECG and PK sample collection and central blinded ECG interval measurement and interpretation. Across all time points at steady state, the baseline-adjusted QTc intervals and the upper bounds of their associated 90% CIs were ≤ 20 ms following 240-mg once-daily doses of apalutamide. Consistent with the primary endpoint results, categorical analysis of absolute QTcF values revealed a slightly higher incidence of QTcF readings > 450 but ≤ 480 ms on C3D1 compared with baseline and C1D1. These results indicate that the QTc increases observed with apalutamide become apparent at steady state (after minimally 4 weeks) but not after the first dose, likely because of an accumulation of apalutamide with repeat dosing.

There were two outliers with a larger QTc prolongation. One patient with a QTcF interval > 480 and ≤ 500 ms also had a higher predose QTcF value (469.3 ms) and had a concurrent C3D1 observation of grade 1 hypocalcemia. A second patient had a large absolute ΔQTcF (60.4 ms) at 1 h post dose on C3D1 (QTcF 455.7 ms), which may have been related to exposure to apalutamide (6.84 µg/mL) or *N*-desmethyl apalutamide (8.21 µg/mL). However, the QTcF value was lower at 2 h post dose despite higher drug concentrations [QTcF of 446 ms (change of 58.3 ms)], with exposure of apalutamide and *N*-desmethyl apalutamide of 8.77 and 8.87 µ/mL, respectively, suggesting that the increase of more than 60.4 ms was not consistent at similar exposure within the same individual. No patients discontinued treatment due to QTc prolongation, and no evidence of development of ventricular arrhythmias was observed that could be attributed to underlying QTc prolongation.

According to Sarapa et al., the magnitude of changes in QTcF interval (as observed in the present study) may be considered a mild to moderate QTc-prolonging effect for an anticancer agent, and these same authors have suggested that a dedicated QTc study for anticancer agents that excludes ∆QTc of < 20 ms can be concluded as a negative study [21], consistent with the present data showing no new clinical concerns [4]. Our data are supported by a small and voluntary QTc substudy of the SPARTAN trial [6], which also revealed no patients in the apalutamide arm with a QTcF interval > 480 ms. In the placebo arm of the SPARTAN study, two of six patients had at least one postdose QTcF interval > 450 and ≤ 480 ms; one of these two patients had a baseline QTcF interval > 450 ms.

De novo T-wave abnormalities were observed in three patients (7%) and were absent at baseline, and no QTcF prolongation ≥ 480 ms was observed in these three patients. No evidence for an apalutamide treatment effect was noted on the length of the PR interval in our study. The observed mean increases in QRS duration as observed on C3D1 were minimal (< 2.5 ms) and are considered not clinically meaningful.

Overall, exposures of apalutamide and its extent of accumulation observed in this study are consistent with those previously reported [7]. To explore the relationship between apalutamide concentration at steady state and QTcF, PK (plasma concentration) and PD (change from baseline in QTcF) data were analyzed using a linear mixed-effects model. The analysis revealed an association between plasma concentration of apalutamide and QTcF and predicted a prolongation of 13.81 ms at *C*_max_ at steady state (C3D1), with an upper bound of two-sided 90% CI of 17.85 ms. Because of the correlation between apalutamide and *N*-desmethyl apalutamide exposures, a similar association between plasma concentration of *N*-desmethyl apalutamide and QTcF was detected. Based on the flat PK profile of *N*-desmethyl apalutamide at steady state, the apparent association between *N*-desmethyl apalutamide concentration and QTcF at steady state is considered less clinically meaningful compared with the parent drug. Overall, results of the PK/PD analysis indicated that a large effect on ∆QTcF is not expected at steady state following 240 mg daily dose of apalutamide.

These data from a Phase Ib QT/QTc study that investigated the effect of apalutamide on QTc intervals revealed no new safety signals associated with apalutamide treatment in men with CRPC. For the primary endpoint, no significant safety findings related to QT prolongation were documented and there were no observed arrhythmias related to apalutamide. Overall, the safety profile observed in this study was as expected based on the known safety profile of apalutamide and results from other studies [6, 7, 19, 20].

Overall, these data demonstrate that apalutamide does not produce clinically meaningful changes in QTc interval or produce a concerning effect on ventricular repolarization in patients with CRPC.
